# Supplementary material for: Universal mechanism for hybrid percolation transitions
Source: Sci Rep. 2017 Jul 18;7:5723. doi: 10.1038/s41598-017-06182-3 (PMC5515945; doi:10.1038/s41598-017-06182-3)
Supplement: Supplementary file 1 — Supplmentary Information [file 41598_2017_6182_MOESM1_ESM.pdf]

Supplemental Information for Universal mechanism for hybrid percolation transitions

Deokjae Lee,[1] Wonjun Choi,[1] J. Kertész,[2,3] and B. Kahng[1,*]

[1] *CCSS, CTP and Department of Physics and Astronomy, Seoul National University, Seoul 08826, Korea*
[2] *Center for Network Science, Central European University, Budapest, Hungary*
[3] *Department of Theoretical Physics, Budapest University of Technology and Economics, Budapest, Hungary*

(Dated: May 7, 2017)

* bkahng@snu.ac.kr

CONTENTS

I. Analytic results for the HPT of the SWIR model 3
A. Transition point 3
B. Condition for the occurrence of the discontinuous transition 4

II. Size and lifetime distributions of the critical branching trees 5

III. Loop-length distribution for the reaction $W + I \to 2I$ 6

IV. The avalanches in k-core percolation 6

V. How to determine effective degrees of each node in the CF model 7

VI. Schematic illustration of the avalanches in the CF model 8

VII. Statistics for the loop-formations in the avalanches in the CF model 9

VIII. Discussion 9

I. ANALYTIC RESULTS FOR THE HPT OF THE SWIR MODEL

A. Transition point

In an absorbing state, each node is in one of three states, the susceptible S, weakened W and recovered R states. We consider the probability $P_S(\ell)$ that a randomly selected node is in state S when it contacts ℓ neighbors in state R. This probability means that the node remains in state S even though it has been in contact ℓ times with those ℓ neighbors in state I before they change their states to R. Thus we obtain

$$P_S(\ell) = (1-\kappa-\mu)^{\ell}, \tag{1}$$

where $\kappa(\mu)$ is the reaction probability of S becoming I(W) by single attack. Next, $P_{\mathrm{W}}(\ell)$ is similarly defined as the probability that a randomly selected node is in state W after it contacts ℓ neighbors in state I before they change their states to R. The probability $P_W(\ell)$ is given as

$$P_W(\ell) = \sum_{n=0}^{\ell-1}(1-\kappa-\mu)^n\mu(1-\nu)^{\ell-n-1}, \tag{2}$$

where ν is the probability of W becoming I by single contact with neighboring I. Finally, $P_R(\ell)$ is the probability that a node is in state R when it contacts ℓ neighbors in state R in the absorbing state. Using the relation $P_S(\ell) + P_W(\ell) + P_R(\ell) = 1$, one can determine $P_R(\ell)$ in terms of P_S and P_W.

The order parameter m that a randomly chosen node is in state R after the system falls into an absorbing state is given as

$$m = \sum_{q=1}^{\infty} P_d(q) \sum_{\ell=1}^{q} \binom{q}{\ell} r^{\ell}(1-r)^{q-\ell}P_R(\ell), \tag{3}$$

where $P_d(q)$ is the probability that a node has degree q and r is the probability that an arbitrarily chosen edge leads to a node in state R in the absorbing state. Using the local tree approximation, we define r_n similarly to r but now at the tree level n.

The probability r_{n+1} can be derived from r_n as follows:

$$r_{n+1} = \sum_{q=1}^{\infty}\frac{qP_d(q)}{z}\sum_{l=0}^{q-1}\binom{q-1}{\ell} r_n^{\ell}(1-r_n)^{q-1-\ell}P_R(\ell) \equiv f(r_n), \tag{4}$$

where the factor $qP_d(q)/z$ is the probability that a node connected to a randomly chosen edge has degree q. As a particular case, when the network is an ER network having a degree distribution that follows the Poisson distribution, i.e., $P_d(q) = z^q e^{-z}/q!$, where $z = \sum_q qP_d(q)$ is the mean degree, the function $f(r_n)$ is reduced as follows:

$$f(r_n) = 1 - \Big(1 - \frac{\mu}{\kappa+\mu-\nu}\Big)e^{-(\kappa+\mu)zr_n} - \frac{\mu}{\kappa+\mu-\nu}e^{-\nu z r_n}. \tag{5}$$

Eq. (4) reduces to a self-consistency equation for r for given reaction rates in the limit $n \to \infty$. Once we obtain the solution of r, we can obtain the outbreak size m using Eq. (3). For ER networks, however, m becomes equivalent to r so that the solution of the self-consistency equation Eq. (4) yields the order parameter. Thus we define $F(m) \equiv f(m) - m$ so that the order parameter satisfies the following equation

$$F(m) = 1 - e^{-(\kappa+\mu)zm}\Big(1 - \frac{\mu}{\kappa+\mu-\nu}\Big) - \frac{\mu}{\kappa+\mu-\nu}e^{-\nu z m} - m = 0. \tag{6}$$

When m is small, the function $F(m)$ is expanded as follows:

$$F(m) \approx am + bm^2 + cm^3 + O(m^4), \tag{7}$$

where

$$a = (\kappa z - 1) \tag{8}$$

$$b = -\frac{1}{2}(\kappa^2 + \kappa\mu - \mu\nu)z^2 \tag{9}$$

$$c = \frac{1}{6}\Big(\kappa^3 + 2\kappa^2\mu + \kappa\mu(\mu-\nu) - \mu\nu(\mu+\nu)\Big)z^3 \tag{10}$$

with the mean degree z. $m = 0$ is a trivial solution of $F(m) = 0$. c is supposed to be negative.

We remark that although non-trivial solutions exist for appropriate values of a and b, some of them might be physically irrelevant for a given initial condition. We can find out the relevancy by checking the stability of the fixed points of $r_{n+1} = f(r_n)$. If we impose a small perturbation to the steady state solution r^* of Eq. (4), we obtain the recursive equation as

$$r^* + \delta r_{n+1} \approx f(r^*) + \left.\frac{df}{dr}\right|_{r=r^*} \delta r_n, \tag{11}$$

which leads to

$$\eta \equiv \frac{\delta r_{n+1}}{\delta r_n} = \left.\frac{df}{dr}\right|_{r=r^*}. \tag{12}$$

If $\eta < 1$ (> 1), then the steady state solution r^* is stable (unstable). Note that $\eta < 1 (> 1)$ iff $F(m^*) < 0 (> 0)$. If $a < 0$, the trivial solution $m = 0$ becomes stable so that other non-trivial solutions cannot be accessible. On the other hand, since $F(1)$ is always negative, the condition $a > 0$ guarantees the existence of a non-trivial solution that is stable and physically accessible. This shows $a = 0$ at the transition point which implies $\kappa_c = 1/z$.

B. Condition for the occurrence of the discontinuous transition

The solution of $F(m) = 0$ within the order of m^3 is $m = 0$ and $m_*^{\pm}$, where

$$m_*^{\pm} = -\frac{b}{2c} \pm \sqrt{\frac{b^2}{4c^2} - \frac{a}{c}}. \tag{13}$$

When a discontinuous phase transition occurs at $a = 0$, i.e., at $\kappa_c = 1/z$, $-b/c$ is still positive. Since $b = 0$ at

$$\kappa_b = \frac{-\mu + \sqrt{\mu^2 + 4\mu\nu}}{2}, \tag{14}$$

and $b < 0$ for $\kappa > \kappa_b$, b has to be positive at κ_a as long as $\kappa_a < \kappa_b$. This is the condition for a discontinuous transition to occur at κ_c with a nonzero order parameter $m_* = -b/c$. The condition is rewritten in another form as

$$\frac{1}{z} < \frac{-\mu + \sqrt{\mu^2 + 4\mu\nu}}{2}. \tag{15}$$

II. SIZE AND LIFETIME DISTRIBUTIONS OF THE CRITICAL BRANCHING TREES

Here we check if the scaling features of $O(N^{2/3})$ and $O(N^{1/3})$ for the size and the lifetime, respectively, of a CB tree at a transition point z_c are valid even for any $z \gg z_c$. We obtain the distributions of the size and the lifetime of the critical branching trees on ER random networks with mean degree $z = 8$ and on fully-connected networks with different system sizes, respectively. Performing numerical simulations with different systems of size N, we obtain that the size distribution of CB trees follows a power law, $p_s \sim s^{-\tau_s} \exp(-s/s^*)$, where $\tau_s \approx 3/2$ and $s^* \sim N^{2/3}$ (see Fig. S1). The life time distribution also follows a power law $p_\ell(\ell) \sim \ell^{-\tau_\ell} \exp(-\ell/\ell^*)$ with $\tau_\ell \approx 2$ and $\ell^* \sim N^{1/3}$ (see Fig. S2). This numerical results are consistent with the analytic results using the generating function method for a CB tree.

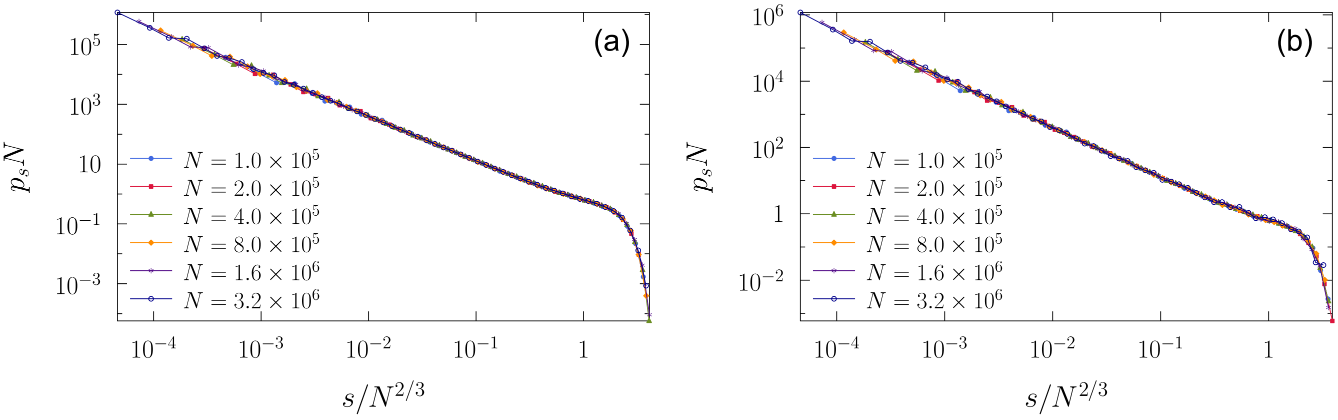

Figure S1. Scaling plot of the size distribution of CB trees on (a) ER networks with mean degree $z = 8$ and (b) fully connected networks with degree $z = N - 1$ for different system sizes. From a randomly selected seed, a branch is constructed to each neighbor with probability $1/z$. Repeatedly each of the offspring makes a branch to their neighbors with the same probability $1/z$. We find for both cases that the size distribution of the tree decays a power-law way with the exponent $\tau_s \approx 3/2$ and there exists a characteristic size $s^* \sim N^{2/3}$ for the CB trees.

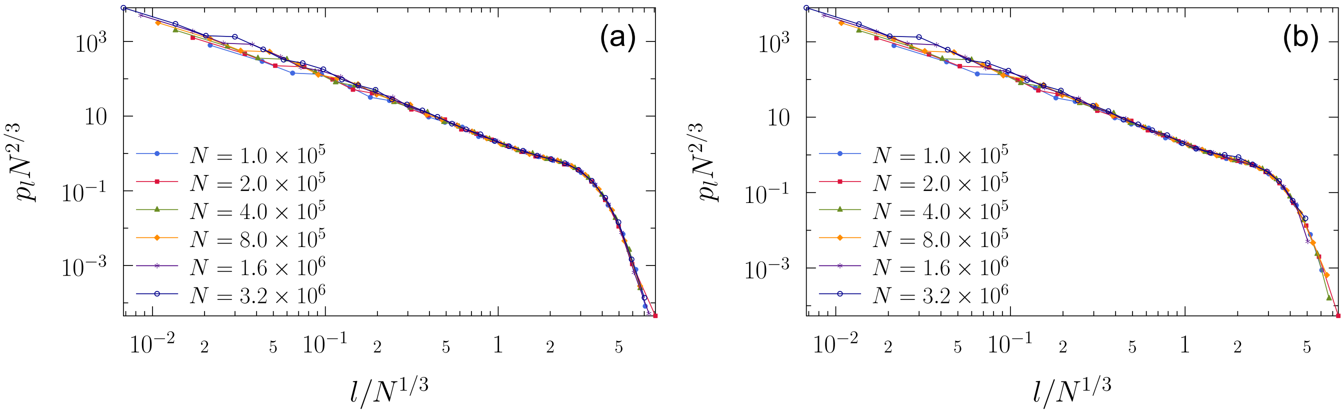

Figure S2. Scaling plot of the lifetime distribution of CB trees on (a) ER networks with mean degree $z = 8$ and (b) fully connected networks with degree $z = N - 1$ for different system sizes. From a randomly selected seed, a branch is constructed to each neighbor with probability $1/z$. Each of the offspring makes a branch to its neighbors with the same probability $1/z$. This process is repeated successively. We find that the lifetime distribution of the tree decays in a power-law way with the exponent $\tau_\ell \approx 2$ and there exists a characteristic size $\ell^* \sim N^{1/3}$ for the CB trees.

III. LOOP-LENGTH DISTRIBUTION FOR THE REACTION $W + I \to 2I$

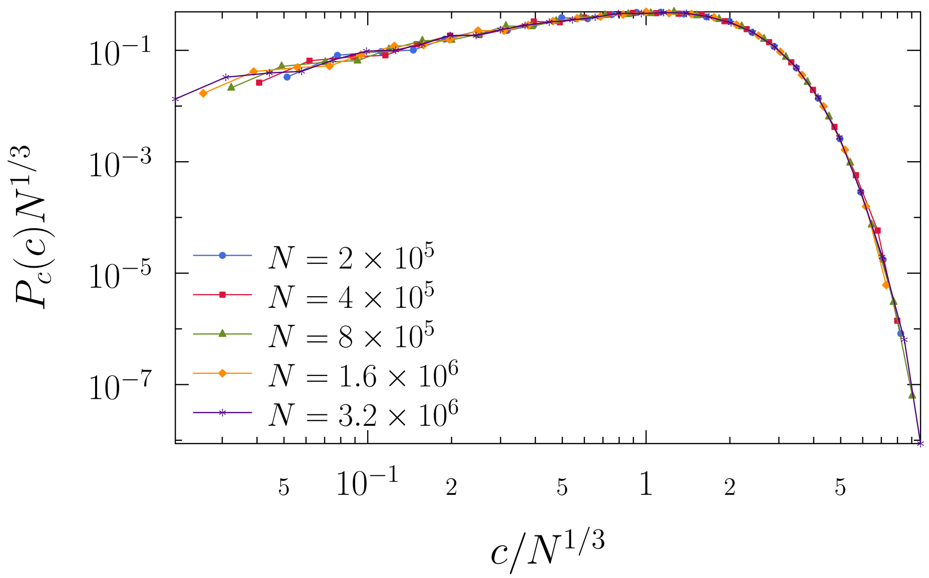

Figure S3. Scaling plot of the distribution $P_c(c)$ of the lengths c of loops (i.e., cycle) that are formed by the reaction $W+I \to 2I$ for different system sizes. The scaling is in the form of $P_c(c)N^{1/3}$ vs $c/N^{1/3}$. Here loop length is defined as one plus the sum of the distances from the generations n_I and n_w to their last common ancestor. The nodes i and j are connected by the reaction $W + I \to 2I$ and make a loop. Data are well collapsed onto a single curve and loop lengths are scaled by a characteristic scale $\sim N^{1/3}$.

IV. THE AVALANCHES IN k-CORE PERCOLATION

The avalanche processes of (k=3)-core percolation is schematically illustrated in Fig. S4. At the beginning, all nodes are classified into the two types, susceptible nodes with degree $k = 3$ (green circles) and generalized weakened nodes with $k > 3$ (dark blue circles). The dynamics proceeds in the following way: In (a), an infinite avalanche process begins at a node with any degree $k \geq 3$ (red circle). Here we take the case $k = 3$. This node corresponds to an infectious seed for the SWIR model. In (b), when that triggering node is intentionally removed (black circle), the degrees of its neighbors are decreased by one. If the degree of a neighbor becomes $k < 3$ (red), $k = 3$ (light blue), and $k > 3$ (dark blue), the node is regarded as an infected node (corresponding to $S + I \to 2I$) and is removed next step (red), is regarded as a weakened (light blue), and remains as a generalized weakened node (dark blue), respectively. In (c), the red node is removed and becomes black. The degree of its neighbor is again decreased by one. If a degree becomes $k < 3$ (red), the susceptible neighbor becomes infected, which is to be removed next step. In (d), the red node in (c) is removed and the degrees of its neighbors are decreased by one. Here the red node, whose degree was originally four, now becomes infected by the contacts with previously two red nodes. Such reaction processes correspond to $S + I \to W + I$ and $W + I \to 2I$. This removal process is possible only when a loop is formed between the light-blue node and the red node in (c). Loop length is shown rather short in this schematic figure but loops can be long as much as $O(N^{1/3})$ in an ER graph. The processes (a)-(d) continue until no more red node remains.

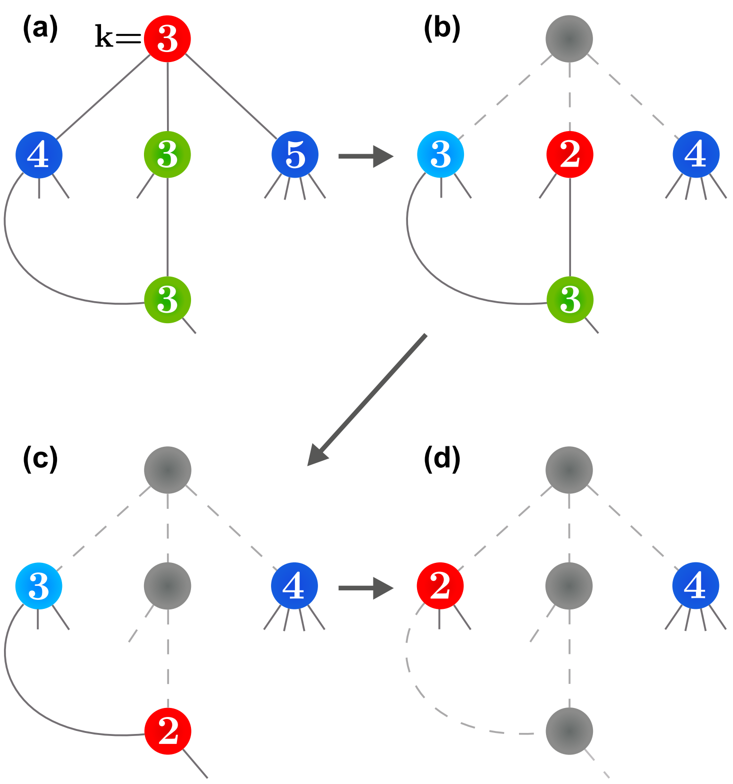

Figure S4. Schematic illustration of an avalanche processes of $(k = 3)$-core percolation. At the beginning, if degree of a node is $k = 3$ $(k > 3)$, the node is represented by green (dark blue) circle, which corresponds to a susceptible (generalized weakened) node for the SWIR model. If degree of a node becomes $k = 3$ during the dynamics, that node is regarded as a weakened node (light blue). The number in each circle represents the degree of that node at respective step.

V. HOW TO DETERMINE EFFECTIVE DEGREES OF EACH NODE IN THE CF MODEL

As defined in the main text, to determine the effective degrees of each node for an infinite avalanche, we needed to count the number of each type of links of a certain node i following which we can access $O(N)$ nodes along only the same type of links. Such links of the node i are called viable links. To implement this counting in finite systems, we present a method at a certain graph with mean degree z.

First, we generate an ER graph with mean degree z for each type of links, then determine a GMCC. Second, to determine the effective degree $k_A(i)$ of a node i for A-type links, we consider a network that consists of all nodes in the GMCC and only A-type of links, which is denoted as A-GMCC. Next, to determine the viable links of a node i in the A-GMCC, we suppose that the node i is removed, and determine an A-GMCC. If this removal does not break the A-GMCC at all, then all A-type links of the node i are viable links of the node i. If the removal breaks A-GMCC into more than one clusters, there may be a unique largest one. Then the links of the node that were connected to the largest cluster are the viable links of type A of the node. One may suppose the case that two or more clusters are of the same size; however, its probability would be negligible. Type B viable links are also determined in the same way. This determination can be efficiently implemented using the algorithms in Refs. [Hwang et al., Phys. Rev. E 91, 022814] and [Holm et al., J. ACM 48, 723].

The avalanche process is implemented straightforwardly with this definition of viable links. First, we obtain effective degrees of each node in the GMCC, and then identify weakened or susceptible state of each node. To trigger an avalanche, we remove a randomly chosen node, which serves as a seed of the avalanche. At the next time step, we recalculate effective degrees of each neighbor of the seed. If there are neighbors whose one or both types of effective degrees are zero, then we remove them. We repeat these processes with all neighbors of the nodes removed at the previous time step. The repeated process continues until no more node is removed.

VI. SCHEMATIC ILLUSTRATION OF THE AVALANCHES IN THE CF MODEL

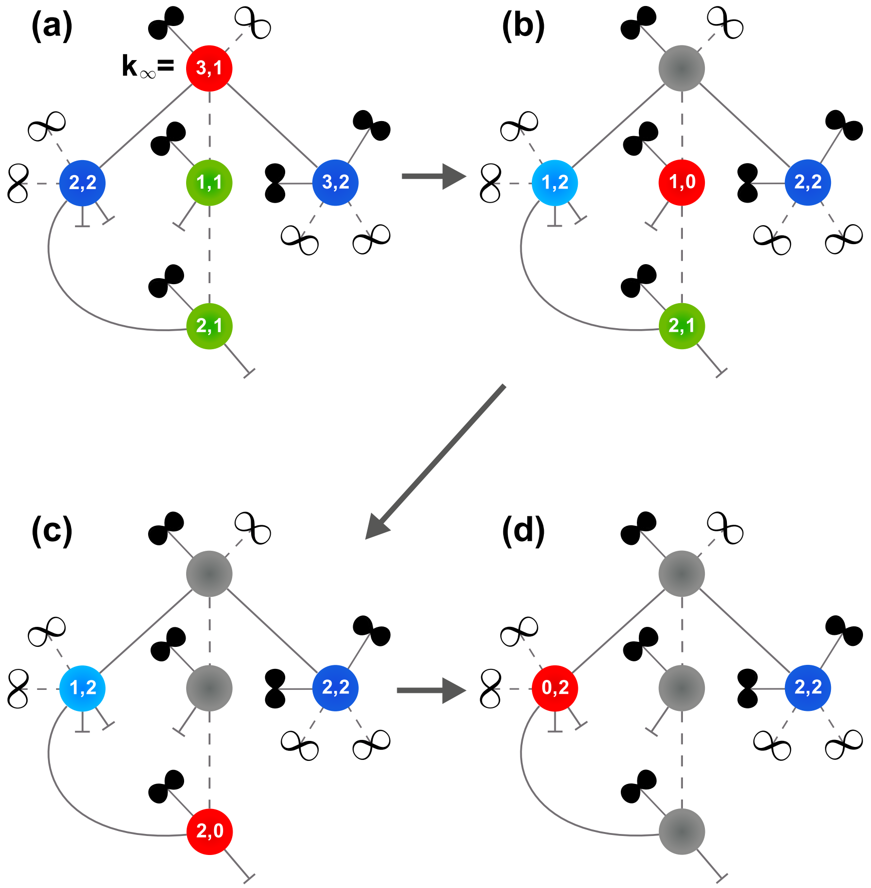

Figure S5. Schematic illustration of an infinite avalanche process in the CF model

There exist two types of links represented by solid and dashed lines. Infinity mark (filled or empty ∞) on a link (solid or dashed) represents that one can reach $O(N)$ number of nodes through the links of that type. Links with bar (|) mean that one can reach only $o(N)$ nodes. Pair of numbers inside the circle of each node represent effective degrees of respective node. Those are the number of solid links and dashed links in order that lead to $O(N)$ number of nodes. If a node loses all such links of any type, it is separated from the GMCC. For instance, the node with the effective degree (1,1) at the middle of Fig.S5(a) is a member of the GMCC through its only solid link and the dashed link connected to the red node.

Nodes with degree one for any type of links are colored by green, which correspond to susceptible nodes. Nodes with degree more than one for any type of links are colored by dark blue, which corresponds to generalized weakened state in k-core percolation. The cascading dynamics starts by removing a randomly selected node in any state in (a), denoted by red circle, which is a seed. Then, the effective degrees of the neighbors of the red node are changed as follows: the degree (2,2) change to (1,2), (1,1) to (1,0) and (3,2) to (2,2). After that, the red node becomes recovered (denoted as black circle). The node with updated degrees as (1,2) in (b) changes its color from dark to light blue, representing that the node now becomes weakened, because it can be infected by contacting one more infected node via the solid line (i.e., losing the green neighbor). The node updated as (1,0) no longer belongs to the GMCC and must be removed next step shown in (c). The red node in (b) is removed. Consequently, the node with effective degree (2,1) in (b) changes to the degree (2,0) and shall be removed next step. The red node in (c) is removed. One of its neighbors with degree (1,2) in (c) changes its degree to (0,2), which is to be removed next step in (d). This corresponds to the reaction $W + I \to 2I$. This reaction is possible through the long-range loop in (c).

VII. STATISTICS FOR THE LOOP-FORMATIONS IN THE AVALANCHES IN THE CF MODEL

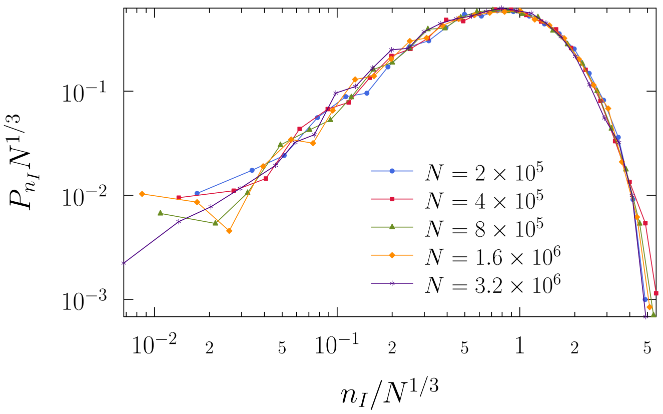

Figure S6. For the CF model, scaling plot of the probability distribution P_{n_I} of the generation n_I at which a node in state W becomes I for the finite avalanche case. Data for different system sizes are well collapsed onto a single curve with the scaling form of $P_{n_I} N^{1/3}$ as a function of $n_I/N^{1/3}$. This means that the loops of length $O(N^{1/3})$ are abundant. This is actually expected because finite size effect arises at the generation $O(N^{1/3})$.

VIII. DISCUSSION

In statistical physics of critical phenomena, universality means that there is a class of systems for which (among others) the critical exponents are the same. There are irrelevant parameters of these systems (e.g., lattice type), which do not lead out of such a universality class and there are relevant ones (e.g., dimensionality), which do. The situation is similar for the studied case. The ER graph belongs certainly in a different universality class than the finite dimensional lattices, but ER graphs with different average degrees behave universally. This can be seen in the derivation of the exponent 1/3 for the size dependence of the characteristic time n_c. In the derivation the mapping to the critical branching process played a pivotal role, where the fact that the underlying topology is an ER graph is important. In finite dimensional lattices there are loops, which destroy the above mentioned mapping. Accordingly, the reaction $W + I \to 2I$ can occur any time, and the weakened nodes are not accumulated. Then the HPT does not occur in k-core and the CF model in finite dimensional lattice below a critical dimension. (It would be an interesting theoretical question to investigate, whether there is a critical dimensionality, above which the ER result could be obtained but this goes beyond the scope of this paper.) The average degree is irrelevant as the system is anyway diluted to criticality, but merely changes the transition point. As we presented earlier, the transition point is given as $\kappa_c = 1/z$.
